# Statistical prediction of immunity to placental malaria based on multi-assay antibody data for malarial antigens

**DOI:** 10.1186/s12936-017-2041-3

**Published:** 2017-09-29

**Authors:** Chathura Siriwardhana, Rui Fang, Ali Salanti, Rose G. F. Leke, Naveen Bobbili, Diane Wallace Taylor, John J. Chen

**Affiliations:** 1 Biostatistics Core, Department of Complementary and Integrative Medicine, John A. Burns School of Medicine, University of Hawaii at Manoa, Honolulu, HI 96813 USA; 2 Centre for Medical Parasitology at Department of Immunology and Microbiology, University of Copenhagen and Department of Infectious Diseases, Copenhagen University Hospital, Copenhagen, Denmark; 3 The Biotechnology Center, Faculty of Medicine and Biomedical Research, University of Yaoundé I, Yaoundé, Cameroon; 4 Department of Tropical Medicine, Medical Microbiology and Pharmacology, John A. Burns School of Medicine, University of Hawaii at Manoa, Honolulu, HI 96813 USA

**Keywords:** Predictive models, Placental malaria, Multiplex assays, VAR2CSA

## Abstract

**Background:**

*Plasmodium falciparum* infections are especially severe in pregnant women because infected erythrocytes (IE) express VAR2CSA, a ligand that binds to placental trophoblasts, causing IE to accumulate in the placenta. Resulting inflammation and pathology increases a woman’s risk of anemia, miscarriage, premature deliveries, and having low birthweight (LBW) babies. Antibodies (Ab) to VAR2CSA reduce placental parasitaemia and improve pregnancy outcomes. Currently, no single assay is able to predict if a woman has adequate immunity to prevent placental malaria (PM). This study measured Ab levels to 28 malarial antigens and used the data to develop statistical models for predicting if a woman has sufficient immunity to prevent PM.

**Methods:**

Archival plasma samples from 1377 women were screened in a bead-based multiplex assay for Ab to 17 VAR2CSA-associated antigens (full length VAR2CSA (FV2), DBL 1-6 of the FCR3, 3D7 and 7G8 lines, ID1-ID2a (FCR3 and 3D7) and 11 antigens that have been reported to be associated with immunity to *P. falciparum* (AMA-1, CSP, EBA-175, LSA1, MSP1, MSP2, MSP3, MSP11, Pf41, Pf70 and RESA)). Ab levels along with clinical variables (age, gravidity) were used in the following seven statistical approaches: logistic regression full model, logistic regression reduced model, recursive partitioning, random forests, linear discriminant analysis, quadratic discriminant analysis, and support vector machine.

**Results:**

The best and simplest model proved to be the logistic regression reduced model. AMA-1, MSP2, EBA-175, Pf41, and MSP11 were found to be the top five most important predictors for the PM status based on overall prediction performance.

**Conclusions:**

Not surprising, significant differences were observed between PM positive (PM+) and PM negative (PM−) groups for Ab levels to the majority of malaria antigens. Individually though, these malarial antigens did not achieve reasonably high performances in terms of predicting the PM status. Utilizing multiple antigens in predictive models considerably improved discrimination power compared to individual assays. Among seven different classifiers considered, the reduced logistic regression model produces the best overall predictive performance.

**Electronic supplementary material:**

The online version of this article (doi:10.1186/s12936-017-2041-3) contains supplementary material, which is available to authorized users.

## Background

*Plasmodium falciparum* infections in pregnant women increases the risk of maternal anaemia, spontaneous abortions, premature deliveries, and low birthweight (LBW) babies, because infected erythrocytes (IE) sequester in the intervillous space (IVS) of the placenta causing placental malaria (PM). Sequestration is mediated by the binding of the malarial antigen VAR2CSA on the surface of IE with chondroitin sulfate A on trophoblasts lining the IVS. Over several pregnancies, women in malaria-endemic areas can produce antibodies (Ab) to VAR2CSA-expressing IE that are associated with improved pregnancy outcomes, e.g., reduced prevalence of maternal anaemia [1, 2], lower placental parasitaemias [3–5], decreased prevalence of LBW babies [6], improved infant birthweights [2, 7] and lower risk of drug-treatment failures [1]. Thus, Ab to VAR2CSA play a significant role in immunity against PM; however, no single Ab test or method is available to determine if a woman has sufficient immunity to prevent PM.

Serological correlates of protection for Ab to VAR2CSA have been sought. VAR2CSA consists of six Duffy Binding-Like (DBL) domains and several interspersed domains [8–10]. Each of the six domains and Ab to full length VAR2CSA (FV2) have been implicated in protection [9–15], but Ab levels to FV2 or a single DBL domain alone is not adequate to determine if a woman is protected from PM. Developers of VAR2CSA-based vaccine have employed functional tests to measure the ability of Ab to inhibit the binding of IE to CSA, but it has been difficult to link inhibition of binding activity with absence of PM in women living in malaria endemic areas. In a high malaria transmission area, the absence of PM at delivery was found to correlate with high Ab levels to FV2 at 5 months of pregnancy (P = 0.005); Ab to multiple DBL domains and allelic variants (P = 0.003), and proportion of high avidity Ab to FV2 (P = 0.0009) [16, 17]. Similarly, in an urban area, when 420 plasma samples from multigravid women collected at delivery screened in 21 serological assays for Ab to FV2, different DBL variants, and Ab avidity to FV2, the only immune parameter that correlated with protection was proportion of high avidity Ab [18]. In the study, a 5% increase in proportion of high avidity Ab was associated with nearly a 15% lower likelihood of PM. Thus, some correlates of protection exist, but none are robust enough to accurately predict the PM immune status of pregnant women.

Analytical tools that help predict if women have acquired protective immunity to PM are needed. The availability of a cost-effective diagnostic approach to identify a woman’s level of immunity would allow (1) doctors to provide better prenatal care, (2) vaccine developers to assess the level of immunity women have before and after vaccination, and (3) government officials to make intelligent health policies for pregnant women. Because immunity to *P. falciparum* infections is complex, few attempts have been made to develop predictive models of immunity [19, 20]. However, since immunity to PM is thought to be mediated by Ab to a single malarial antigen, it may be possible to develop predictive models. This study evaluates the hypothesis that using a comprehensive multiple-assay approach will lead to improved predictive power. In the current study, plasma collected at delivery from 1377 pregnant women with and without PM were screened in 28 serological assays for IgG to FV2 and different lines of the six DBL domains, as well as, eleven Ab to antigens associated with malarial immunity (AMA-1, CSP, EBA-175, LSA1, MSP1, MSP2, MSP3, MSP11, Pf41, Pf70 and RESA). Multivariable logistic regression and five other statistical classification methods were used to determine the optimal number of assays, along with other clinical variables such as age and gravidity, to provide the best prediction model.

## Methods

### Ethical approvals

De-identified, archival plasma samples used in the current study were exempt from human subject research by the Committee on Human Studies, University of Hawaii, Manoa (CHS#21891). The original study was approved by the National Ethics Committee, Cameroon and the Institutional Review Board at Georgetown University, and participants gave written informed consent to use their blood to study Ab to malaria.

### Study design and plasma samples

Archival plasma samples from a cross-sectional study conducted between 1996 and 2001 [21, 22] in Yaoundé, Cameroon were used. Details on study site, enrollment criteria, sample collection, and laboratory tests are reported in Tako et al. [22]. Yaoundé is a malaria endemic area where the entomological inoculation rates were estimated to be 13 infectious bites per person per year at the time the samples were collected [23, 24]. In the initial study, pregnant women were enrolled at the Biyem-Assi Hospital, a district hospital that mainly cares for women in the surrounding area, and the Central Maternity Hospital, a referral hospital for a diverse group of women [22]. An advantage of using clinical data and plasma from this cohort is that the study was conducted before implementation in 2004 of intermittent preventive treatment (IPT). Thus, women in the study developed naturally-acquired immunity to PM that aided in clearing their placental infections. Post-IPT implementation, placental parasitaemias are prevented by the use of sulfadoxine–pyrimethamine, making it impossible to assess the role of naturally acquired immunity. Women were not tested for HIV; however, the prevalence of HIV among pregnant women attending antenatal-clinics in Yaoundé at the time was 4.0–13.6% [25], making it unlikely HIV infections had a major impact on the study.

Women were enrolled at delivery and information about the mother (age, gravidity, obstetric history, length of pregnancy, and use of chemoprophylaxis during pregnancy) and the newborn (e.g., birthweight) was recorded on a standardized questionnaire. Maternal peripheral blood, IVS blood, and a biopsy of placental IVS tissue were collected.

### Laboratory tests

Thick and thin blood smears were prepared of maternal peripheral and IVS blood, and impression smears were made of biopsied placental tissues. Slides were stained with Diff-Quick and slides were read by two microscopists. Capillary tubes were filled with maternal peripheral blood and the percent pack cell volume (PCV) determined following centrifugation. Placental biopsies were fixed in 10% buffered formalin, embedded, stained with haematoxylin–eosin, and examined for parasites. A woman was considered to be PM positive (PM+) if IE were detected in either IVS blood smears, impression smears of villous tissue, or histological section of the placenta. Women were considered to PM negative (PM−) if parasites were not detected in peripheral and placental smears. Women with peripheral parasitaemia, but without placental parasitaemia, were excluded from study. Women were considered anemic if they had < 30% PCV, as defined by the WHO. Singletons weighing less than 2500 grams were considered LBW, and those delivered between 28 and 37 weeks of gestation were classified as being pre-term.

### Sample selection

All women with PM (n = 341 PM+) and delivered live neonates ≥ 28 weeks of gestation were included. For comparison, approximately three times of the number (n = 1036) of PM− women were randomly selected who met the inclusion criteria.

### Recombinant proteins

Recombinant proteins used in this study have been reported previously [16–18] and included full-length FV2 (FCR3 line), DBL1 + 2, ID1-ID2a (the minimal CSA binding site of VAR2CSA), individual domains DBL1 through DBL6 (3D7, 7G8 and FCR3 lines). In addition, 11 known non-VAR antigens (AMA-1, CSP, EBA-175, LSA1, MSP 1, MSP2, MSP3 and MSP11, Pf41, Pf 70 and RESA) were tested. Detailed information on the antigens has been previously published [26].

### Preparing multiplex of recombinant proteins coupled to microspheres

The method for coupling recombinant proteins to SeroMAP microspheres has been described previously [16, 27]. In brief, the optimal amount of each protein for saturating 1 million microspheres was covalently coupled to beads with different spectral addresses overnight at 4 °C. Following blocking with phosphate buffered saline (PBS) containing 1% bovine-serum albumin (PBS-1% BSA), the microspheres were multiplexed by pooling equal numbers of each antigen to create three pools of 9-10 antigens each. The pools include 1) Multiplex R (9 antigens)—DBL1 (IT4 line), ID1-ID2a (3D7 line), DBL1 + 2 (FCR3 line), DBL4 (7G8 line), DBL5 (3D7 line), DBL6 (7G8 line), MSP1, MSP3, MSP11; 2) Multiplex S (10 antigens)—DBL1 (7G8 line), 1D1-ID2a (FCR3 line), DBL2 (FCR3 line), DBL3 (7G8 line), DBL5 (FCR3 line), DBL6 (FCR3 line), LSA1, AMA-1, Pf41, RESA; and 3) Multiplex T (9 antigens)—FV2 (FCR3 line), DBL1 (3D7 line), DBL3 (FCR3 line), DBL4 (FCR3 line), DBL5 (7G8 line), CSP, Pf70, EBA-175, MSP2. Seven controls were included on each plate: pools of (1) plasma from pregnant American women, (2) Cameroonian males, (3) two dilutions of plasma from multigravid Cameroonian women (4 wells) and PBS.

### Multi-analyte platform (MAP) assay for IgG Ab

The MAP assay was performed as previously described [16, 17, 27]. In brief, 50 µl of each antigen pool (containing 2000 microspheres of each antigen) were incubated with 50 µl of a 1:200 dilution of plasma in PBS-1% BSA in pre-wetted filter plates (96 well Multiscreen BV; Millipore) for 1 h at 25 °C on a rotating shaker at 650 rpm. Microspheres were washed twice with PBS-0.05% Tween20 and once with PBS-1% BSA. Then, 100 µl of secondary Ab [R-phycoerythrin-conjugated, Affini Pure F(ab′)_2_ fragment, goat anti-human IgG Fc fragment-specific, Jackson Immunoresearch] diluted to 2 µg/ml in PBS-1% BSA was added to each well and incubated as above in the dark for 1 h. Microspheres were washed as described above, re-suspended in 100 µl PBS-1% BSA, and the microsphere suspension was analysed using a Liquichip M100 reader (Luminex Corp., Austin, TX). The reader was programmed to read a minimum 100 beads per spectral address, DD Gate 7500–15,000 and 35 s timeout. The results were expressed as median fluorescence intensity (MFI). The serological assays were repeated twice and the average of the two MFIs was used for analysis.

### Statistical analysis

Demographic, clinical, and assay variables were first summarized using descriptive statistics: means with standard deviations (SD) or median with 25th and 75th percentiles for continuous variables based on distribution; frequencies and percentages for categorical variables, e.g., maternal anaemia status. Two-sample t-tests or Wilcoxon-Rank-Sum test for continuous variables (based on the distribution) and Chi square tests for categorical variables were used to compare women with and without PM. To help visualize the antibody data, we also generated a heatmap, a graphical representation of data, along with clustering of antibodies and patients based on hierarchical clustering (Additional file 1: Figure S1). The associations between the outcome variable, PM status, and each of the assay variables were then evaluated through univariate logistic regression. The area under the Receiver Operating Curve (ROC), or AUC, was calculated to assess the discrimination power of each variable.

**Fig. 1:**
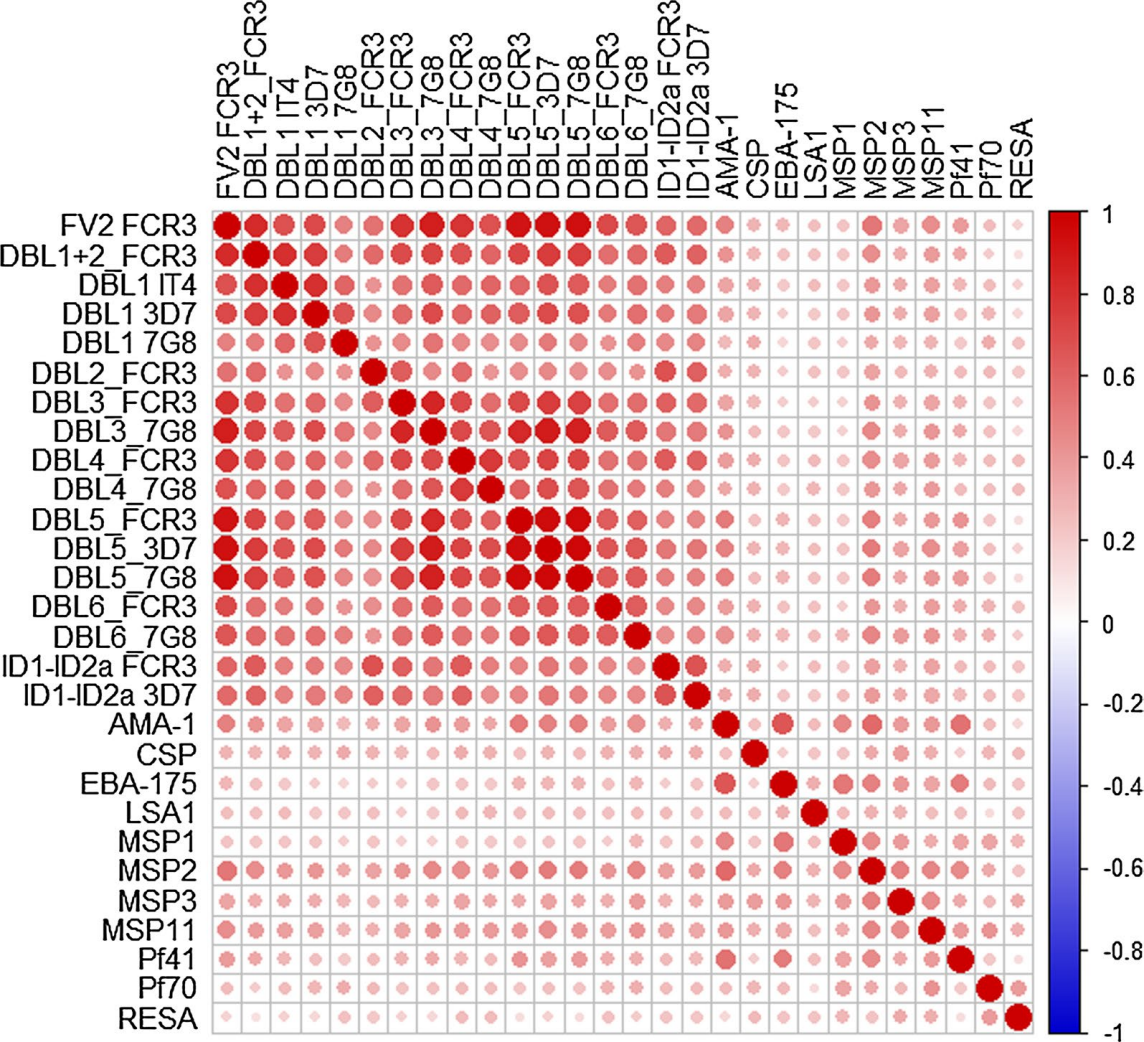
A graphical representation for correlations among antibodies

Next, a suitable subset of demographic and biological assay variables was determined for the statistical predictive models. The inclusion of highly correlated predictor valuables could result in unreliable and unstable estimates for a parametric model. The Pearson correlation coefficients were estimated between pairs of all possible candidate predictive variables. Only one variable between a pair of candidate predictors was selected into the proposed predictors set, if the estimated Pearson correlation coefficient between the pair was larger than a threshold value of 0.8. For example, different DBL domains from the three *P. falciparum* lines, 3D7, 7G8, and FCR3, were highly associated (see Fig. 1). Therefore, only FCR3 was selected for the multivariable model development. Clinical variables with significant amount of missing values were not included in the multivariable analysis. A logistic regression model was first fitted for the malaria status using the proposed set of candidate predictors by incorporating all possible two-way interactions. Next, a stepwise adding and dropping technique [28] was utilized to obtain a reduced model that has a lower Akaike Information Criterion (AIC), for better performance and simplicity. Note that, the model that holds the lowest AIC attains the highest likelihood integrated with a penalty for the number of estimated parameters. The same process was also taken using a different model selection criterion, the Bayesian Information Criterion (BIC), which resulted in the same final model, Performances of proposed multi-assay logistic models were evaluated on multiple performance measures such as overall prediction accuracy, sensitivity, specificity, and AUC. A model that archived large values of the above measures can be reasonably considered as a “good” model for the binary classification problem.

To avoid model overfitting, the classification performance was assessed by applying the fivefold cross validation technique. Initially, the dataset of 1377 individuals was partitioned into five approximately equal-sized random subgroups or folds. Four of the five folds were used for model development, while the remaining fold was used as a test set to examine the performance of the classification. This procedure was repeated 500 times with different sets of random partitions to evaluate the average performance. Here we determine the optimal probability cut-point for class specification by maximizing the Youden’s J statistic, which is a commonly applied tool in binary classification problems for optimizing both sensitivity and specificity.

To compare with the above logistic regression approach, other classification techniques, i.e., classifiers, for developing predictive models were also considered. For this purpose, five approaches namely Recursive Partitioning (RPART), Random Forests (RF), Linear Discriminate Analysis (LDA), Quadratic Discriminant Analysis (QDA), and Support Vector Machine (SVM) were considered [29–34]. These five classifiers were selected due to the diverse nature of their underlying techniques.

A classification tree is used for classifying an observation into a certain class among multiple choices, based on the values of predictor variables. The algorithm develops a tree type structure to discriminate an observation based on its characteristics, by determining optimal cut points for each predictive variable to specify a class. The results are easy to interpret and visualize and can potentially uncover patterns in the data that cannot be easily identified through traditional regression methods. The recursive partitioning (RPART) [29] is a non-parametric multivariable classification technique that develops a decision tree based on a sequence of logical conditions to determine subsets from a sample based on covariate specification. In this way, RPART allows for a data-driven exploration of non-linear relationships and interactions among many explanatory variables. Algorithms given by CART and C4.5 are two well-known techniques for developing such a classifier [29, 32]. Random forest (RF) [31] is another tree based classifier, but it takes an additional step forward by growing a collection of trees other than a single decision tree. RF is developing by using a set of bootstrap samples randomly drawn from the original data with replacement. About a two-third of data that selected from bootstrapping are used to growing each classification tree, and the remaining one-third of the data called Out-Of-Bag (OOB) are used to obtaining unbiased estimates of classification errors and variable importance. The predicted class of a new observation is determined as the aggregated majority vote by all the decision trees.

Linear Discriminate Analysis (LDA) is a classical classification technique originally proposed for the two class classification [30]. Later, this method was generalized for the multiclass classification [33]. LDA assumes that the class specific attribute variables are multivariate normally distributed with a class specific mean vector and a common covariance matrix shared between classes. Assuming a prior probability for an observation to be of a given class, a posterior probability is then calculated, based on observed data. The most appropriate class for the observation is determined as the one with the highest posterior probability. Quadratic Discriminant Analysis (QDA) is the generalized version of LDA, which relaxes the assumption of shared covariance matrices. Unlike LDA and QDA, Support Vector Machine (SVM) is a supervised machine learning algorithm that does not use distributional assumptions. It seeks hyper planes in a multi-dimensional space of attribute variables that specify decision boundaries. For an optimal solution, the algorithm determines class boundaries with the maximum margin of difference. SVM is known to be one of the broadly improved classification techniques in recent past [34].

In addition to reporting the prediction performance of each of the individual classifier, the agreement between classification decisions was evaluated with respect to the overall prediction, and PM−, PM+ group specific predictions, by analysing the proportion of consensus of classified cases shared by each pair of classifiers. If two classifiers share a large proportion of commonly classified subjects, it ostensibly reflects a high agreement.

An analysis was conducted to further study the importance of proposed predictive variables on predicting PM for the reduced logistic regression model. The investigation was performed parallel to the fivefold cross validation technique, i.e., partitioning the data into five equally sized random folds, with four folds for modelling the classifier and the remaining fold as a test set. Suppose two sets given by *S*_*i*+_ and *S*_*i*−_ represent ith covariate values observed for PM+ and PM− cases, respectively, based on whole data. In the test set, *i*th covariate values of PM+ cases were replaced by a sample drawn from *S*_*i*−_, using the simple random sampling with replacement. In the same fashion, corresponding covariate values of test PM− cases were replaced by a sample drawn from *S*_*i*+_. This mechanism allows one to examine the classifier’s prediction performances under miss-specified *i*th predictor. If the overall predictability, sensitivity, or specificity changes considerably by the permutation approach, the particular variable must be playing an important role in specifying PM status. This method was adopted to quantify the importance of each proposed predictor, by averaging the absolute difference between un-permuted and permuted performance measures, using 1500 random sets of partitions for each predictor. Based on these quantities, rankings of the predictors were calculated with respect to each of the four performance measures. Finally, a common rank list for the proposed predictors was obtained based on the weighted rank aggregation concept [35, 36], using the Spearman’s foot-rule distance, assuming all four performance measures are equally important.

All statistical analyses described were conducted using SAS and R statistical software packages. A *P* value of less than 0.05 was considered as statistically significant.

## Results

### Characteristics of the study sample

The characteristics of the pregnant women and their neonates studied are described in Table 1. Women with PM were younger (P < 0.0001) and had fewer pregnancies (P < 0.0001) compared with those without PM. Women with PM had a higher prevalence of pre-term deliveries (P = 0.014), anaemia (P < 0.0001), and low birth weight babies (P = 0.0011) compared to PM− women. PM+ women also had shorter lengths of pregnancy (P = 0.0006) and lower birth weight babies (P < 0.0001).

**Table 1:** Characteristics of pregnant women and their neonates

Characteristic	PM+ (n = 341)	PM− (n = 1036)	P-value
Age in year, mean (SD)	24.3 (5.4)	26.3 (5.8)	< 0.0001
Gravidity, mean (SD)	2.7 (1.9)	3.2 (2.1)	< 0.0001
Placental parasitaemia by impression smears in %, mean (SD)	5.4 (12.4)	0 (0)	n/a
Clinic location, n (%)			0.59
BA-all	194 (56.9%)	607 (58.6%)	
CM-NHR	147 (43.1%)	429 (41.4%)	
Pre-term delivery, n (%)*	75 (24.2%)	171 (17.8%)	0.014
Length of pregnancy in weeks, mean (SD)	38.3 (3.2)	39.0 (3.0)	0.0006
Intrauterine growth restriction	17 (5.0%)	41 (14.0%)	0.43
Maternal anaemia at delivery, n (%)*	90 (26.4%)	136 (15.3%)	< 0.0001
Haematocrit in %, mean (SD)	31.3 (6.2)	34.9 (5.9)	< 0.0001
Low birth weight babies, n (%)*	70 (21.6%)	139 (14.0%)	0.0011
Baby weight in grams, mean (SD)	2922 (656)	3134 (635)	< 0.0001

For variables with missing values indicated by * sign, percentages are calculated by non-missing data

### Summary of antibody assay variables between PM+ and PM− women

The descriptive statistics of assay variables between PM+ and PM− women are shown in Table 2. PM+ women recognized more DBL domains (P < 0.0001) and had higher IgG levels to FV2, DBL1 + 2, DBL2, DBL3, DBL4, DBL5, DBL6 (all lines, all P < 0.0001) and ID1-ID2a (3D7 line, P < 0.0001). In addition, women with PM had higher IgG levels to AMA-1, CSP, EBA-175, LSA1, MSP1, MSP2, MSP3, MSP11, Pf41, Pf70 (all P < 0.0001) and RESA (P = 0.0095). The predictive accuracy of each variable was assessed by determining the AUC using the ROC method based on the entire data set. Table 2 summarizes the AUCs for individual predictors. The discriminative powers of each individual assay were between 55 and 70% range. AMA-1 and MSP2 had the best individual predictive performance.

**Table 2:** Comparisons of antibody levels

Variable	PM+ (n = 341)	PM− (n = 1036)	P-value	AUC
FV2 FCR3	4820 (1360, 11,497)	1427 (426, 5553)	< 0.0001	0.66
DBL1 + 2 FCR3	2195 (746, 5944)	829 (277, 3034)	< 0.0001	0.65
DBL1 IT4	1261 (421, 3130)	476 (201, 1320)	< 0.0001	0.66
DBL1 3D7	1258 (511, 3037)	571 (225, 1433)	< 0.0001	0.65
DBL1 7G8	921 (392, 1925)	583 (249, 1250)	< 0.0001	0.60
DBL2 FCR3	815 (410, 1737)	526 (189, 1275)	< 0.0001	0.60
DBL3 FCR3	1286 (434, 4193)	625 (148, 2334)	< 0.0001	0.62
DBL3 7G8	2126 (603, 6850)	717 (201, 3190)	< 0.0001	0.65
DBL4 FCR3	2096 (617, 4960)	875 (229, 2757)	< 0.0001	0.63
DBL4 7G8	1108 (384, 3383)	451 (161, 1522)	< 0.0001	0.64
DBL5 FCR3	7987 (793, 19,534)	1031 (138, 10,278)	< 0.0001	0.66
DBL5 3D7	3898 (620, 10,411)	718 (164, 5251)	< 0.0001	0.66
DBL5 7G8	4940 (743, 12,478)	686 (178, 6776)	< 0.0001	0.66
DBL6 FCR3	1277 (393, 3686)	494 (150, 1648)	< 0.0001	0.64
DBL6 7G8	4389 (1766, 9280)	2274 (793, 5222)	< 0.0001	0.64
ID1-ID2a FCR3	474 (212, 1475)	304 (120, 773)	< 0.0001	0.61
ID1-ID2a 3D7	2089 (884, 3952)	1356 (490, 3243)	< 0.0001	0.58
Number of domain recognized out of 6	2 (0, 3)	0 (0, 2)	< 0.0001	0.64
Number of variants recognized out of 13	3 (0, 6)	0 (0, 4)	< 0.0001	0.65
AMA-1	23,769 (17,479, 24,375)	14,621 (3597, 23,680)	< 0.0001	0.70
CSP	398 (193, 891)	231 (137, 545)	< 0.0001	0.62
EBA-175	17,755 (4388, 24,143)	5263 (731, 20,562)	< 0.0001	0.66
LSA1	452 (158, 1833)	190 (71, 736)	< 0.0001	0.63
MSP1	4851 (1045, 13,335)	1856 (450, 8148)	< 0.0001	0.61
MSP2	11,432 (3751, 22,159)	3303 (805, 10,511)	< 0.0001	0.69
MSP3	1069 (362, 4188)	413 (149, 1853)	< 0.0001	0.63
MSP11	1629 (629, 4031)	681 (298, 1789)	< 0.0001	0.66
Pf41	5214 (809, 17,004)	1654 (239, 8324)	< 0.0001	0.63
Pf70	815 (470, 1478)	602 (294, 1093)	< 0.0001	0.60
RESA	84 (49, 228)	73 (43, 179)	0.0095	0.55

The data were summarized by median MFI with 25th and 75th percentiles

AUC was based on univariate logistic regression models

P-value was based on Wilcoxon-Rank-Sum test

### Logistic multi-assay predictive models

**Fig. 2:**
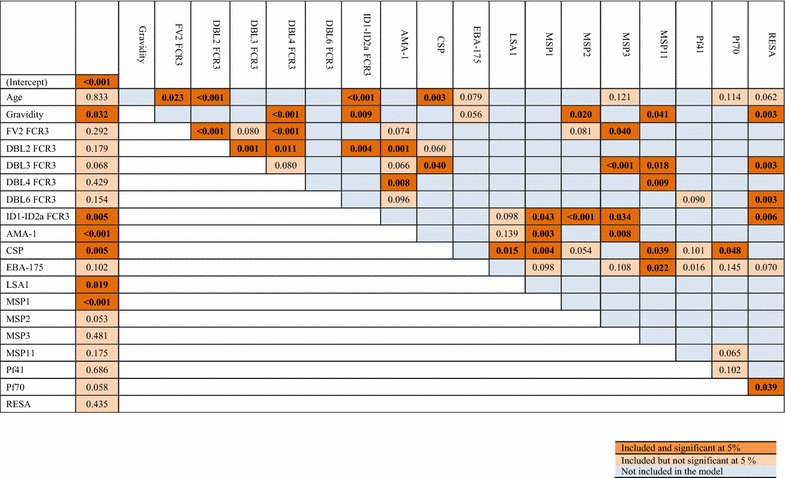
A graphical representation of individual predictors and interactions included in the final logistic regression (LR-Reduced) model. First column indicates predictor’s main effect significance and remaining columns presents the pairwise interactions integrated in the model along with significance. Note that interactions are presented only once

The study hypothesized that a combination of assays would improve discriminatory power, as each assay would contribute somewhat unique information to a predictive model. Two multivariable logistic regressions models were generated using demographic variables and individual assays with their pairwise interactions. A complete logistic regression model (LR-Full) was first developed using a set of nineteen individual predictive variables and their all possible pairwise interactions. A reduced version of it was then developed by utilizing an “adding-and-dropping” criteria to achieve a more simplified model with the minimum AIC value [28]. Figure 2 illustrates the resulted components along with their significance for the reduced logistic regression model (LR-Reduced). Among nineteen individual predictors used in the model, six variables namely ID1- ID2a (FCR3 line), AMA-1, CSP, LSA1, MSP1, and gravidity were reported to have significant main effects. Suggesting the potential complex paths of controlling PM, many associations appeared to be significant for the model. For instance, a wide set of significant pairwise interactions between mothers’ age with other factors were observed.

### Comparison of the seven predictive models based on full data and cross-validation

In addition to the two logistic regression models, five other classification algorithms (RF, RPART, LDA, QDA, and SVM) were also used to develop predictive models. Prediction performances information of all seven classifiers with respect to the four types of performance measures are presented in Table 3, both based on the full data of 1377 individuals and through cross-validation.

**Table 3:** Prediction performances of seven classifiers on discriminating PM+ and PM−, evaluated using the fivefold cross validation technique and full data

Classifier	Overall Accuracy	Specificity	Sensitivity	AUC
LR-Full	0.68	0.51	0.73	0.64
LR-Reduced	0.71	0.66	0.73	0.76
RPART	0.69	0.54	0.74	0.67
RF	0.75	0.30	0.90	0.74
LDA	0.45	0.82	0.33	0.73
QDA	0.49	0.63	0.46	0.71
SVM	0.51	0.42	0.83	0.69

*LR-Full* Logistic Regression Full model, *LR-Reduced* Logistic Regression Reduced model, *RPART* Recursive Partitioning, *RF* Random Forests, *LDA* Linear Discriminant Analysis, *QDA* Quadratic Discriminant Analysis, *SVM* Support Vector Machine

Based on the fivefold cross-validation approach, RF holds the highest accuracy approximately 75%, and next LR-Reduced with approximately 71% overall accuracy in predicting the PM. These two were the best amongst all seven classification methods with respect to overall accuracy measure. Overall accuracies by other methods ranged from 45 to 69%. Based on the accuracy of predicting protection from PM (PM−, sensitivity), RF clearly outperformed all other classifiers, holding about 90% sensitivity. In general, except LDA and QDA, all other classification methods showed reasonably good performance on the sensitivity. Although RF had a better performance in terms of overall accuracy and the sensitivity, it had the worst performance on (i.e., about 30%) predicting infection of PM (PM+, specificity). LDA and LR-Reduced were the top two classifiers with respect to the specificity. For a binary classifier, higher values of AUC reflect its balanced performance accounting for both sensitivity and specificity. Calculated AUC’s of seven classifiers ranged from 64 to 76% with LR-Reduced being the best. Considering the overall performance assessment by AUC, the LR-Reduced is recommended as the most appropriate classifier for predicting PM, on the proposed set of demographic and biological predictors. This model holds reasonably good sensitivity and specificity measures. Although LR-Reduced model that had approximately 0.76 AUC, we noticed that the AUC for AMA-1 alone using the univariate logistic regression models was as high as 0.7. Therefore, extensive prediction performance boost by the multivariate approach was not observed as hypothesized.

### Agreement of classifiers

These classifiers’ predictions were further investigated by evaluating the agreement on their classification decisions. As shown in Table 4, from the prospect of overall classification, consensus decisions among classifiers ranged from 42 to 86%, reflecting diverse prediction attributes. Similar results were observed for PM− and PM+ prediction cases. Approximately consensus decisions rates were changed from 89 to 41 and 77 to 44%, respectively for PM− and PM+ predictions.

**Table 4:** Agreements among seven classifiers discriminating an individual, evaluated using the fivefold cross validation technique

	LR-Full	LR-Reduced	RPART	RF	LDA	QDA	SVM
LR-Full							
(a)	1.00	0.85	0.76	0.73	0.53	0.51	0.71
(b)	1.00	0.87	0.79	0.77	0.52	0.51	0.74
(c)	1.00	0.77	0.66	0.63	0.58	0.53	0.62
LR-Reduced							
(a)	0.85	1.00	0.76	0.72	0.57	0.53	0.73
(b)	0.87	1.00	0.79	0.77	0.53	0.52	0.75
(c)	0.77	1.00	0.67	0.58	0.70	0.58	0.65
RPART							
(a)	0.76	0.76	1.00	0.86	0.49	0.50	0.76
(b)	0.79	0.79	1.00	0.89	0.48	0.49	0.80
(c)	0.66	0.67	1.00	0.77	0.54	0.51	0.64
RF							
(a)	0.73	0.72	0.86	1.00	0.42	0.47	0.76
(b)	0.77	0.77	0.89	1.00	0.41	0.47	0.80
(c)	0.63	0.58	0.77	1.00	0.44	0.47	0.64
LDA							
(a)	0.53	0.57	0.49	0.42	1.00	0.82	0.54
(b)	0.52	0.53	0.48	0.41	1.00	0.84	0.52
(c)	0.58	0.70	0.54	0.44	1.00	0.74	0.61
QDA							
(a)	0.51	0.53	0.50	0.47	0.82	1.00	0.52
(b)	0.51	0.52	0.49	0.47	0.84	1.00	0.51
(c)	0.53	0.58	0.51	0.47	0.74	1.00	0.55
SVM							
(a)	0.71	0.73	0.76	0.76	0.54	0.52	1.00
(b)	0.74	0.75	0.80	0.80	0.52	0.51	1.00
(c)	0.62	0.65	0.64	0.64	0.61	0.55	1.00

Based on (a). overall classification, (b). PM− classification, and (c). PM+ classification

### Importance of variables in prediction performance

Based on the permutation approach described in the methods section, the importance of each variable of the LR-Reduced model in each performance measure is summarized in Table 5. This includes absolute deviations calculated between before and after permuting each candidate predictor and corresponding rankings with respect to performance. Here, larger values of absolute deviation indicate crucial variables upon the specific performance measure used. For example, MSP2, AMA-1, EBA-175, MSP11, and ID1-ID2a (FCR3) were the top five important predictors that have an impact on the accuracy of correctly detecting PM+ cases. Based on the rank aggregation [35, 36] over all four performance measures, the top five important antibodies affecting the prediction performance were identified as AMA-1, MSP2, EBA-175, Pf41, and MSP11.

**Table 5:** Absolute deviations between overall accuracies reported for un-permuted and permuted cases, by each predictor variable for the final logistic model (LR-Reduced)

Predictor	Overall accuracy	Specificity	Sensitivity	AUC
Age	0.001 (18)	0.001 (18)	0.002 (15)	<0.001 (19)
Gravidity	0.002 (17)	0.001 (19)	0.002 (14)	0.003 (13)
FV2 FCR3	0.007 (08)	0.011 (06)	0.005 (11)	0.006 (09)
DBL2 FCR3	0.003 (13)	0.008 (10)	0.001 (18)	0.009 (06)
DBL3 FCR3	0.002 (15)	0.007 (11)	0.001 (16)	0.002 (14)
DBL4 FCR3	0.007 (07)	0.007 (12)	0.007 (08)	0.001 (17)
DBL6 FCR3	0.012 (05)	0.009 (09)	0.013 (05)	0.009 (07)
ID1-ID2a FCR3	0.005 (09)	0.011 (05)	0.004 (12)	0.001 (16)
AMA-1	0.036 (01)	0.028 (02)	0.039 (01)	0.032 (01)
CSP	0.003 (14)	0.010 (08)	0.001 (17)	0.003 (12)
EBA-175	0.017 (02)	0.018 (03)	0.017 (02)	0.019 (03)
LSA1	0.005 (11)	0.002 (17)	0.006 (10)	0.001 (18)
MSP1	0.005 (12)	0.003 (15)	0.007 (07)	0.002 (15)
MSP2	0.017 (03)	0.029 (01)	0.013 (04)	0.021 (02)
MSP3	<0.001 (19)	0.004 (13)	<0.001 (19)	0.004 (11)
MSP11	0.010 (06)	0.013 (04)	0.009 (06)	0.011 (05)
Pf41	0.014 (04)	0.010 (07)	0.016 (03)	0.011 (04)
Pf70	0.002 (16)	0.004 (14)	0.002 (13)	0.004 (10)
RESA	0.005 (10)	0.003 (16)	0.006 (09)	0.007(08)

Values inside brackets provide ranks of each variable. Rank one is given to the variable with the highest absolute deviation

## Discussion

To our knowledge, this is the first comprehensive step towards the identification of correlates of protection for PM using a multi-assay approach. Different combinations of immunological assays measuring immunity to VAR2CSA and non-pregnancy antigens in PM+ and PM− Cameroonian women were used to derive the best predicted model. The principal findings are that multiple assays can lead to improved power compared to the univariable approach using a single assay.

In the construction of multivariable logistic regression models, a larger model that included a set of nineteen potential demographic and biological predictors and their pairwise interactions was first estimated. Then, a final reduced model was determined using a stepwise approach. The final model demonstrated a moderate improvement in predicting the PM status given mothers’ information, compared to the univariable approach, indicating the advantages of utilizing multiple predictors as outlined in objectives of this study. As alternatives to the logistic regression approach, five other types of commonly used classifications techniques were considered. Several comparative studies evaluating classifiers performances showed diverse predictive capabilities with respect multiple performance measures. When the global performances of classifiers were considered across multiple attributes such as overall accuracy, sensitivity, specificity, and AUC, the logistic regression approach can be reasonably considered the best for this classification problem. In addition to developing predictive models, a permutational method was applied to examine the importance of each predictive variable for the reduced logistic regression model, upon different attributes. This technique is a general concept that can be applied to any parametric or nonparametric classifier in finding rankings of importance of predictors. Furthermore, the top five ranked predictors that are crucial on all four attributes considered were identified.

Since antibody responses data generally follow a highly-skewed distribution, the classifiers were also trained using the log transformed antibody data, considering that such approach may improve the robustness of parameter estimates by diminishing the effects of extreme data points. However, generally, the prediction performance was low with this approach. In addition, the impact of adding second order polynomials of antibodies was also evaluated comparing with the corresponding univariate models, but the predictive performances were not largely improved. There are several potential limitations of the current study. The true nature of the natural acquisition of immunity to placental malaria during the pregnancy is unclear without longitudinal data. No sufficient information is available to evaluate the influence of HLA on the current study, but based on available data and that the women had similar ethnic backgrounds, the influence is likely to be minimal. It is likely that women in the study were infected several times during pregnancy, as the samples were collected at the time of delivery. Selection of the threshold value for the maximum correlation allowed between pairs of predictors was based on the general consideration of minimizing the multicollinearity. A separate test set was not used for validation, but instead utilized the standard K-cross validation (i.e., with K = 5) technique to evaluate the performance of a classifier, which provides an unbiased estimate of performance measures compared to the conventional approach that uses a single fixed test set. The averaging via many random folds removes the biasedness that could cause by the partition(s) selection. It is also important to note that, the use of an independent test set that comes from an entirely a different population than the study population may provide a strong evidence for a validation, but such data with the exact same measurements measured in a substantially large number of subjects with similar inclusion criteria was not available for an extensive study. Although the permutational approach is a sensible way of examining the importance of predictors, one can follow any other suitable alternatives based on his/her objectives. When the rank aggregation approach was applied to determine a commonly important set of predictors, all four performance measures were equally prioritized, which can be also conducted with different weights for each performance measures using a reasonable weighting criterion. Despite these limitations, the current study demonstrated the potential utility of incorporating multiple malarial antigens in developing predictive models for PM states.

Predictive models use data to find associations between a specific outcome, in this case presence/absence of PM at delivery, and other host and immune parameters, but do not always identify factors involved in protection. In this study, the top five important variables affecting prediction performance were having Ab to the non-VAR antigens, AMA-1, MSP2, EBA-175, Pf41, and MSP11. The results suggest that Ab to key merozoite antigens are important, which is logical, since a previous study conducted in Yaoundé found that half of women who were malaria-positive during the second trimester lacked Ab to FV2 at delivery, but were PM−. Thus, Ab to antigens other than VAR2CSA appear to be important in eliminating PM. Overall, the key predictors identified using the modeling approach are logical.

## Conclusion

Improved discrimination ability of PM status compared to individual assays was observed when multiple malarial antigens were considered together. Based on a comparison study, the reduced logistic regression approach was found to have the overall best predictive quality with respect to multiple performance measures, compared with several other commonly used classification approaches for developing predictive models. Additionally, the antigens’ impact on multiple performance measures in the final model was quantified and the top PM status predictors were identified as AMA-1, MSP2, EBA-175, Pf41, and MSP11.

## Additional file

**Additional file 1.** A heatmap that illustrates the observed antibody levels, along with clustering among antibodies (dendrogram in right) and 1377 patients (dendrogram on top). Below the top dendrogram, the panel with red and blue vertical bars represents malaria infected (in red) and none infected (in blue) subjects. Note that log-transformed antibody levels was used for the ease of visualization.
