# Supplementary figures and images for: Statistical prediction of immunity to placental malaria based on multi-assay antibody data for malarial antigens
Source: Malar J. 2017 Sep 29;16:391. doi: 10.1186/s12936-017-2041-3 (PMC5622501; doi:10.1186/s12936-017-2041-3)

**
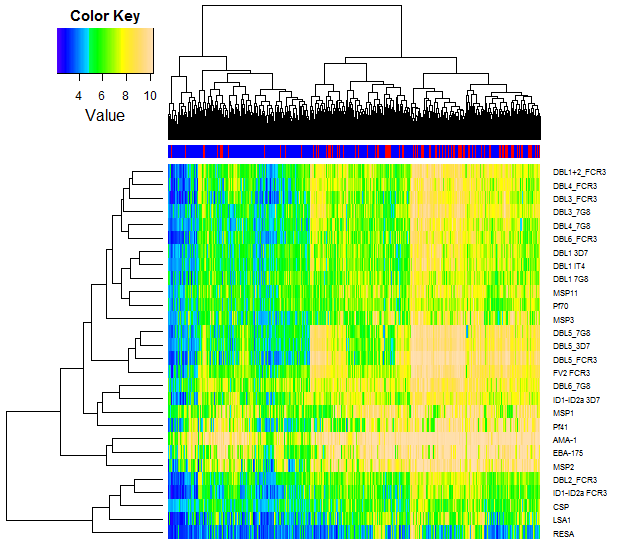
**

Supplement: Supplementary file 1 — Additional file 1. A heatmap that illustrates the observed antibody levels, along with clustering among antibodies (dendrogram in right) and 1377 patients (dendrogram on top). Below the top dendrogram, the panel with red and blue vertical bars represents malaria infected (in red) and none infected (in blue) subjects. Note that log-transformed antibody levels was used for the ease of visualization. [file 12936_2017_2041_MOESM1_ESM.docx]
